# Early detection of local SARS-CoV-2 outbreaks by wastewater surveillance: a feasibility study

**DOI:** 10.1017/S0950268823000146

**Published:** 2023-02-01

**Authors:** Maarten Nauta, Oliver McManus, Kristina Træholt Franck, Ellinor Lindberg Marving, Lasse Dam Rasmussen, Stine Raith Richter, Steen Ethelberg

**Affiliations:** 1 Department of Infectious Disease Epidemiology & Prevention, Statens Serum Institut, 5 Artillerivej, 2300 Copenhagen S, Denmark; 2 European Programme for Public Health Microbiology Training (EUPHEM), European Centre for Disease Prevention and Control (ECDC), Gustav III:s Boulevard 40, 16973 Solna, Sweden; 3 Department of Virus & Microbiological Special Diagnostics, Statens Serum Institut, 5 Artillerivej, 2300 Copenhagen S, Denmark; 4 Department of Public Health, Global Health Section, University of Copenhagen, Øster Farimagsgade 5, 1014 København K, Denmark

**Keywords:** COVID-19, early warning, modelling, wastewater-based surveillance

## Abstract

Wastewater surveillance and quantitative analysis of SARS-CoV-2 RNA are increasingly used to monitor the spread of COVID-19 in the community. We studied the feasibility of applying the surveillance data for early detection of local outbreaks. A Monte Carlo simulation model was constructed, applying data on reported variation in RNA gene copy concentration in faeces and faecal masses shed. It showed that, even with a constant number of SARS-CoV-2 RNA shedders, the variation in concentrations found in wastewater samples will be large, and that it will be challenging to translate viral concentrations into incidence estimates, especially when the number of shedders is low. Potential signals for early detection of hypothetical outbreaks were analysed for their performance in terms of sensitivity and specificity of the signals. The results suggest that a sudden increase in incidence is not easily identified on the basis of wastewater surveillance data, especially in small sampling areas and in low-incidence situations. However, with a high number of shedders and when combining data from multiple consecutive tests, the performance of wastewater sampling is expected to improve considerably. The developed modelling approach can increase our understanding of the results from wastewater surveillance of SARS-CoV-2.

## Introduction

Worldwide, wastewater-based epidemiology (WBE) is increasingly used as a tool to monitor the spread of COVID-19 in the community. The method has proven to be successful in describing epidemiological trends by identifying and quantifying the virus RNA in wastewater samples [1–12]. Additionally, as increasing trend in wastewater may be found prior to those identified by individual testing, it is proposed to be useful for early warning [13–17]. Especially when human testing is limited, it has the potential to predict an increase in the hospitalisation rate, allowing for rapid intervention against local spread of the virus [18].

Before the start of the COVID-19 pandemic, WBE has successfully been applied for several purposes, including surveillance for poliovirus [19]. In a situation where the virus is not circulating in the population, such as in the case of the poliovirus, emergence of the virus can be signalled by wastewater sampling, and wastewater surveillance has proven to be a useful strategy for early detection and intervention [20]. This is particularly useful when samples are obtained at a local scale, so immediate and targeted action can be taken [6, 18]. Research at local or institutional scale (such as in university dormitories) shows that the application of wastewater surveillance is a useful strategy in a situation where re-emergence of SARS-CoV-2 has to be detected in an early stage [21, 22]. However, in the current situation where the epidemic is ongoing and is expected to develop as an endemic disease [23, 24], such elimination and re-emergence may not be a realistic scenario in catchment areas that cover populations with thousands of people or more. In a low-incidence situation, it may be more important to detect increases in incidence that indicate the start of a local outbreak, which should be targeted for a local intervention, before the outbreak spreads further.

For the analysis of wastewater sampling data, it is important to understand the relationship between the number of gene copies (as found by qPCR) and the number of infected people in the population. It has been estimated that between 40 and 67% of infected people shed the SARS-CoV-2 virus in their faeces [25–27], but the timing of faecal shedding remains largely unknown. Using a Monte Carlo simulation model describing the relation between the infection prevalence and the total number of SARS-CoV-2 RNA copies in wastewater, Ahmed *et al*. [1] estimated the number of infections based on Australian wastewater data. Medema *et al*. [12] established a similar theoretical relationship between the number of shedders and the expected virus concentration in wastewater. In their Monte Carlo simulation, they found that the uncertainty of the virus concentration estimated from the number of shedders is dominated by the variation in viral concentrations between people and is particularly large with low numbers of shedders. Whereas Medema *et al*. [12] assumed no decay of the RNA in the sewer signal, McMahan *et al*. [28] incorporated the effects of viral decay over time in their model for viral concentrations in the sewer shed, and applied it in a susceptible-exposed-infectious-recovered model to describe the course of the epidemic.

In Denmark, wastewater surveillance for SARS-CoV-2 has been set up in the course of 2021, and has been organised with samples taken three times a week at 230 locations covering more than 85% of Danish addresses. Population sizes of the catchment areas range between 670 and 638 000 inhabitants (median 10 600). This specific surveillance programme has prompted the need to assess the possible application of wastewater surveillance for early detection of local outbreaks in a low-prevalence situation, which would allow fast local intervention to prevent a wider spread of the virus. To our knowledge, the feasibility of this specific application of wastewater surveillance data has not been studied previously.

In this paper, we therefore explored the possibility for setting up an early warning system for local SARS-CoV-2 outbreaks in a low-prevalence situation, by developing a Monte Carlo simulation model of viral shedding in a wastewater system and analysis of wastewater sampling. As outbreaks are characterised by an increase in the number of infected people, the aim of the modelling was to evaluate the performance of different potential signals for an increase in incidence, based on measured RNA copy counts in two consecutive periods. The results of the modelling may pave the way for the implementation of a systematic routine calculation of potential signals from a wastewater surveillance programme. Although the Danish surveillance programme inspired our study, we used a generic approach for which the conclusions should be valid internationally.

## Methods

### Model description

Based on [12], the relation between RNA copy concentration in wastewater and the number of people shedding the virus in the wastewater system (shedders) can be given as:1
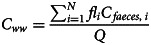
where *C_ww_* is the concentration in the wastewater (gene copies (gc))/l), *N* is the number of infected people shedding the virus, *fl_i_* is the amount of faeces shed by one infected individual *i* (g faeces per person per day), *C_faeces,i_* is the number of gene copies per gram faecal matter shed by infected individual *i* (gc/g faeces) and *Q* indicates the daily water flow to the sewer (l per day).

It follows that the change in log concentration in the wastewater over two measurements is2
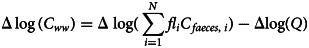
which simplifies to:3
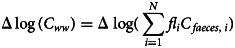
if *Q* does not change between measurements at a specific sampling point.

Given that, by definition,4
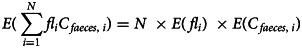
it follows that the relation between the change in log concentration of gene copies in the wastewater, Δlog (*C_ww_*), is expected to be proportional to the change in the log number of shedders, Δ log (*N*), for large values of *N*. Yet, if the variation in *C_faeces,i_* and *fl_i_* between shedders is large and *N* is small, this assumption of proportionality may not be justified. Therefore, published data on *C_faeces_* and *fl* were compared to obtain feasible distributions for these variables in the model.

As qPCR analyses are not perfect, an additional source of variation is added to the values of log (*C_ww_*). This is implemented as *ɛ* ~ Normal(0, *s*_PCR_), where it is assumed that *s*_PCR_ = 0.15 log_10_ units, equivalent to 0.5 Ct value in the qPCR [29, 30].

Therefore, the change in concentration in the wastewater can be obtained from5
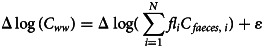


This equation was implemented in a Monte Carlo simulation model, developed in R 4.0.4., where *N* values of *fl_i_* and *C_faeces,i_* are sampled from the distributions given above (see Supplementary Material). The model was used to illustrate the expected dynamics in the observed values of *C_ww_* and to explore the expected performance of potential signals that can be used to identify an increase in incidence. The incidence is assumed to be proportional to the number of infected people shedding the virus (*N*). In the simulations, viral concentrations in human faeces and the amount of faeces shed are assumed to be independent from each other and independent by time. Also, the catchment size is not explicitly included in the model; the wastewater samples are assumed to be taken from well-homogenised wastewater.

### Potential signals

The model was used to assess how wastewater surveillance data may be used to signal a twofold, fourfold or tenfold increase in incidence between two consecutive periods. Based on the model, we explored two potential signals: (a) the difference in the mean of the log *C_ww_* found between two sets of consecutive samples; (b) the *P* value of a linear regression through two sets of consecutive samples.

In (a), the mean of *k* = 3 consecutive samples (1 week in the Danish surveillance programme) is compared with the mean of the next *k* = 3 consecutive samples (the next week). The difference between the two means of the log(*C_ww_*) is determined, and evaluated as a potential signal defined as an increase of more than *D* log units. As a twofold increase implies an increase of 0.3 logs, we evaluated *D* = 0, 0.3, 0.6, 0.9 and 1.2. The same analysis is done for *k* = 6, which may refer to the comparison of two consecutive 2-week periods.

In (b), assuming *k* = 3 consecutive samples per week, a linear regression is performed through the 2 × 3 = 6 data points expressed as log concentrations. The *P* value associated with the slope of an increasing regression line being different from zero, which readily follows from the analysis, is used as a potential signal, defined as *P* < 0.05, *P* < 0.1 or *P* < 0.2. The same analysis is done for *k* = 6, which corresponds to the comparison of two consecutive 2-week periods.

Performance was expressed as sensitivity and specificity of the signal, as obtained from the simulations. Here, the sensitivity is the expected relative frequency in which you get a signal given an increase of the number of shedders between the sets of samples. The specificity is the expected relative frequency in which you do not get a signal given that the number of shedders is unchanged. In the simulations, it was assumed that the number of shedders instantaneously increased from one week to the next, i.e. *N* shedders for the first *k* data points and 2*N*, 4*N* or 10*N* for the second *k* data points. This is a hypothetical scenario that should be identified by a potential signal.

## Results

### Model inputs

Tables 1 and 2 show values and distributions that have been reported for the concentration of viral RNA copies in the faeces, *C_faeces_* and the faecal mass shed per day, *fl*. They show that most authors have used the data presented by [31] (on *C_faeces_*) and [32] (on *fl*). Based on these data, we use the following lognormal distribution for both parameters, as baseline in our analyses:3
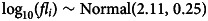
4
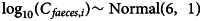

**Table 1.** Reported distributions of the concentration of RNA copies (gene copies, gc) in human faeces (*C_faeces_*)

Study	Distribution of concentrations	Comment
Wölfel *et al*. [31]	Stool samples occur in a range between below LoD to about 7.5 *log_10_ copies per swab* Average 6.76 × 10^5^ *copies per throat/nasal swab*; max 7.11 × 10^8^	Observations from hospitalised patients Decline in concentration over time
Ahmed *et al*. [1]	Uniform (2.56, 7.67) *log_10_ gc/g*	Based on [31]
Curtis *et al*. [34]	Between 100 and 7.1 × 10^8^ *gc/g*	Based on [31]
Lui *et al*. [43]	Between 3 and 6.2 *log_10_ gc/ml* Median 4.1	Observations from hospitalised patients
Han *et al*. [44]	Between LoD and 10.3 *log_10_ gc/ml* Median 7.63	Observations from mildly symptomatic and asymptomatic children
Medema *et al*. [12]	Gamma (10.78, 0.46) mean = 4.95, s.d. 1.5 *log_10_ gc/g*	Based on five publications
Miura *et al*. [41]	Mean 3.4 (95% CrI 0.24–6.5) *log_10_ gc/g* over the whole shedding period Peak concentration 6.5 (95% CrI 5.6–7.4) *log_10_ gc/g*	Based on [31] and a fitted dynamical model for decline over time
Chavarria-Miró *et al*. [45]	Between 3 and 7 *log_10_ gc/g* Mean first 10 days 5.3, later 4.9	Based on [31]
Li *et al*. [46]	Between 1 and 8 *log_10_ gc/g* Mean: 4.23, s.e. 0.133	From systematic review
Schmitz *et al*. [47]	Between 5.74 and 8.28 *log_10_ gc/g* Mean: 7.39, s.e. 0.67 Median: 6.64	Observations from student dormitories
McMahan *et al*. [28]	Normal (7.6,0.8) *log_10_ gc/g faeces* after 5 days; Normal (3.5,0.35) *log_10_ gc/g faeces* after 25 days	Based on [31] Dynamical model includes change in concentration over time

LoD, limit of detection.

**Table 2.** Reported distributions of the daily faecal mass shed by humans (*fl*)

Study	Faecal mass (g) per person per day	Comment
Rose *et al*. [32]	Median 128, mean 149, range 51–796 g wet mass	
Burns *et al*. [48]	Between 105 and 140	
Ahmed *et al*. [1], McMahan *et al*. [28]	Normal (2.11, 0.25) log_10_ units	Based on [32]
Curtis *et al*. [34]	Triang (51, 128, 786)	Based on [32]
Medema *et al*. [12]	Exp^Normal (5,0.58)^ (mean 149, median 126)	Based on [32]
Chavarria-Miró *et al***.** [45]	380	

These distributions are in line with what has been used by others, and have the advantage that the log_10_ values are normally distributed, so some basic statistics apply. Note that the precise mean values, 2.11 and 6, are not important for our approach, as we focus on the change in log concentrations in the wastewater Δlog *C_ww_*. Critical values for our analyses are the standard deviations as given in the equations above.

### Simulation of the number of shedders and the concentration in the wastewater

**Fig. 1.**
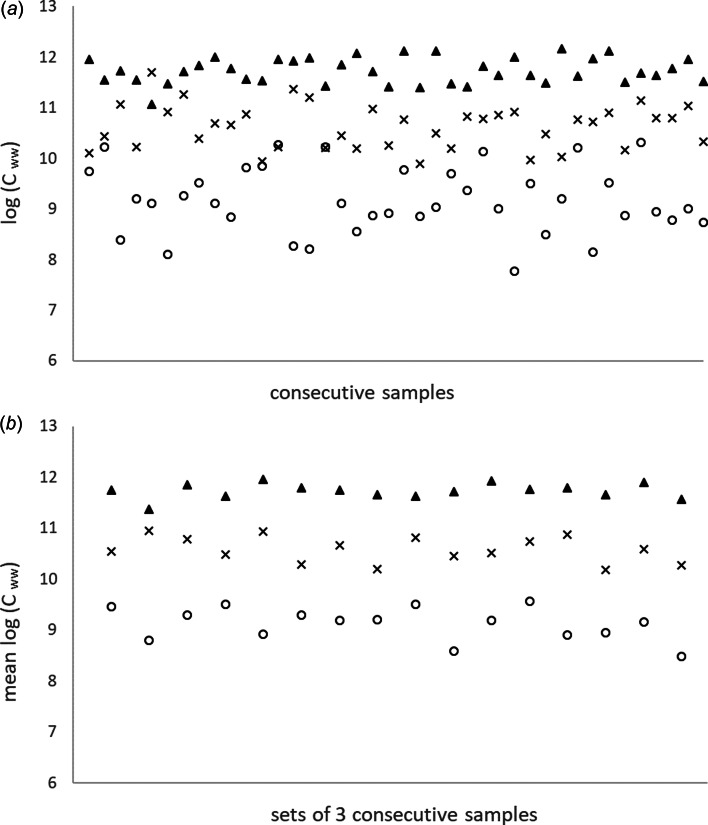
Example of the variation in the observed viral concentration in wastewater *C_ww_* (log gene copies per litre per day) due to random variation in the shedding of virus RNA in a simulation with *N* = 3 (circles), *N* = 30 (crosses) and *N* = 300 (triangles) shedders. (a) Forty consecutive single samples. (b) Consecutive means of independent sets of three samples. The horizontal axis can be taken to represent time, for example, daily independent measurements.

First, the simulation model was run to explore the variation in *C_ww_* as a function of *N*. Figure 1a presents an example of a simulation of a series of 40 measurements over time where the number of shedders is held constant. This illustrates that, on average, the gene copy counts will be higher with a larger number of shedders, and also that the variation in gene copy counts will be substantial. With 100 000 iterations of the simulation model, for *N* = 3, 30 and 300 shedders, means in log (*C*_ww_) are 9.1, 10.6 and 11.8 and standard deviations 0.74, 0.39 and 0.24, respectively. The 95% probability intervals obtained from the 2.5 and 97.5 percentiles are 7.7−10.6, 10.0–11.5 and 11.3–12.3, respectively. Hence, the feasible ranges of observed concentrations overlap, despite the tenfold differences in numbers of shedder. This suggests that individual measurements are unreliable as indicators for an increase in the number of shedders, especially when the number of shedders is low. Figure 1b shows that the mean of log (*C_ww_*) of three samples performs better as an indicator of the number of shedders. The mean log (*C_ww_*) values are the same and the variation is still considerable (standard deviations 0.43, 0.23 and 0.14 with *N* = 3, 30 and 300 shedders respectively), especially when the number of shedders is low. However, the 95% probability intervals (8.3–10.0, 10.2–11.1 and 11.5–12.1 respectively) do no longer overlap.

**Fig. 2.**
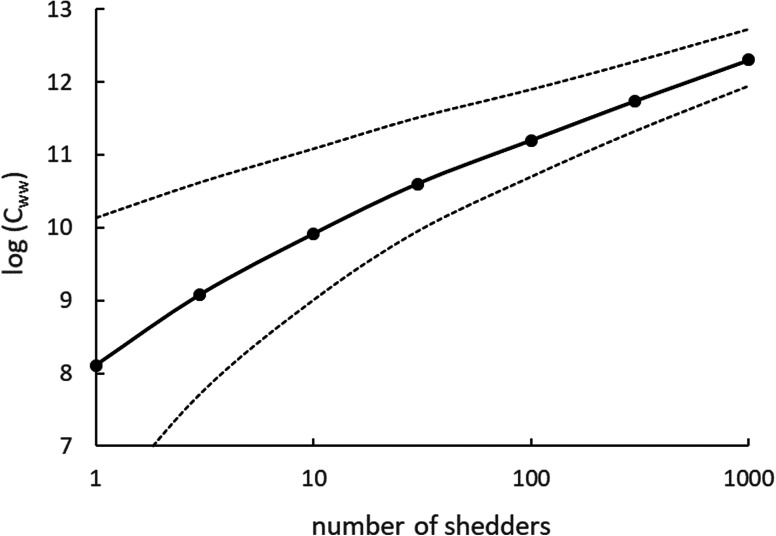
The simulated relation between the number of shedders *N* and the gene copy concentration in the wastewater *C_ww_* (median, 5% and 95% percentiles). Note that both are expressed on a log scale.

Figure 2 illustrates the relation that was obtained between log (*N*) and log (*C_ww_*) and is very similar to one published by [12]. It confirms that the variation between measurements is expected to be large. It also shows that the relation between the mean values of log (*N*) and log (*C_ww_*_)_ is not linear when the number of shedders is low, due to the nature of the lognormal distribution [33].

**Fig. 3.**
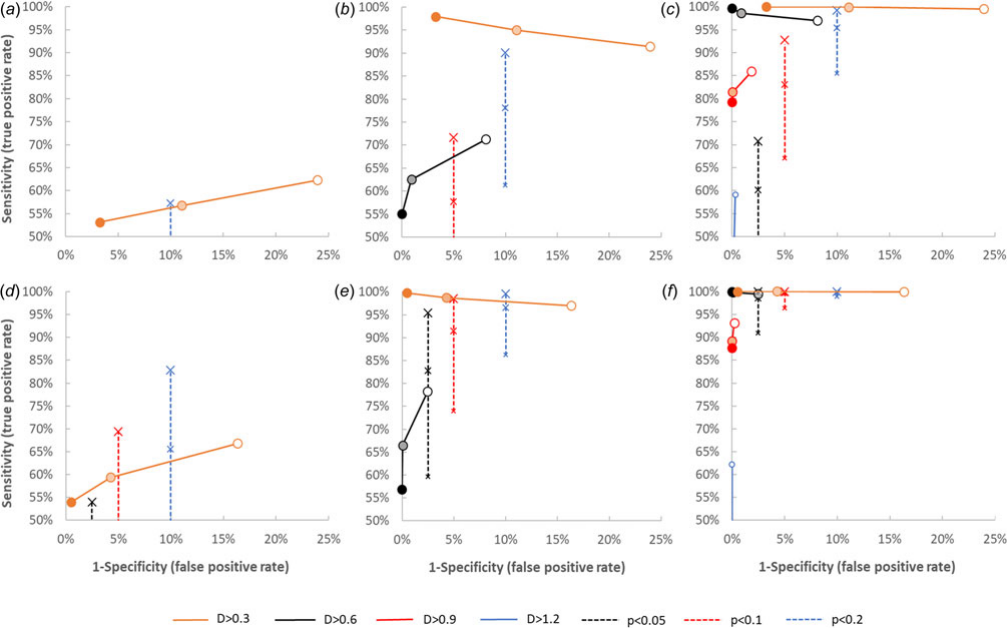
Simulated sensitivity and specificity of potential signals in six scenarios comparing a two- (a, d), four- (b, e) and tenfold (c, f) increase of the number of shedders between two sets of *k* = 3 (a, b, c) and *k* = 6 (d, e, f) samples. Axes correspond to those used for ROC (receiver operating characteristic) curves, only results with sensitivity >50% and specificity >75% are shown. Circles show results for signals based on a difference of means (d), crosses for signals based on linear regression. Open circles/small crosses: *N* = 10; shaded circles/medium crosses: *N* = 100; closed circles/large crosses: *N* = 1000.

Next, the simulation model was used to explore the performance of potential signals by analysis of the frequency of signals without a change in the number of shedders *N*, and with a twofold, fourfold and tenfold increase of *N*. Results are presented in Figure 3, which shows that the performance for all signals is poor for the detection of a twofold increase in *N*, but progressively better for the detection of a fourfold and tenfold increase, especially when two 2-week periods (*k* = 6) are compared. With a fourfold increase, the best performance is from the signal *D* > 0.3log, with initially *N* = 1000 shedders. For a tenfold increase, the *D* > 0.6 signal performs best, with (almost) 100% sensitivity and specificity with initially *N* = 1000 shedders. In general, a higher number of shedders *N* increases the performance of signals, especially if the *D* value is smaller than the log increase in *N*. With an initial number of *N* = 10 shedders, the only signal with sensitivity and specificity >95% is *D* > 0.6 log with a tenfold increase in shedders and *k* = 6.

Interestingly, with the linear regression method, the specificity of the signal is one minus half the *P* value: for *P* < 0.05, the specificity is 0.975, for *P* < 0.1 it is 0.95, etc., because we only look at increasing trends. Here an increased number of shedders always increases the sensitivity of the method.

### Impact of standard deviations

**Fig. 4.**
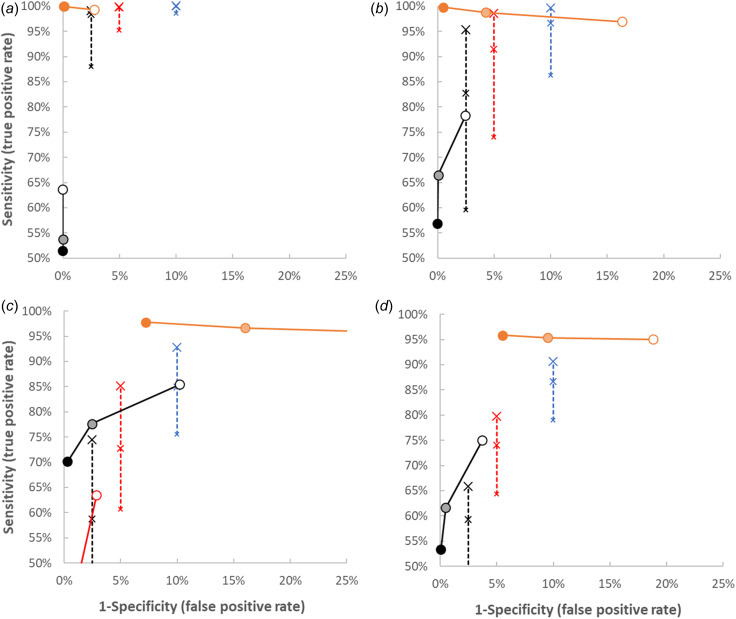
Simulated sensitivity and specificity of potential signals in the scenario with fourfold increase of the number of shedders and two sets of *k* = 6 samples with *σ_faeces_* = 0.5 (a), *σ_faeces_* = 1 (b), *σ_faeces_* = 1.5 (c), *s*_PCR_ = 0.3 (d) and other values as in the baseline. Only results with sensitivity >50% and specificity >75% are shown. Symbols and lines are identical to those in Figure 3.

The performance of potential signals was not affected by the mean values for log (*fl_i_*) and log (*C_faeces,i_*), but was influenced by the standard deviations. This is illustrated in Figure 4, which shows the performance of potential signals for values of the standard deviation of log (*C_faeces,i_*), *σ_faeces_* = 0.5, *σ_faeces_* = 1 and *σ_faeces_* = 1.5, as well as the inherent variation of the qPCR, *s*_PCR_ = 0.3 log_10_ units (equivalent to 0.5 Ct value in the qPCR), for a fourfold increase in *N* and *k* = 6. Similar results are obtained in scenarios with two- and tenfold increase of *N* and/or *k* = 6 (results not shown). These results indicate that with lower standard deviation, the performance increases, whereas it decreases with larger standard deviation.

## Discussion

In this study, we have modelled the expected viral concentrations obtained from RT-qPCR measurements of SARS-CoV-2 in community wastewater samples, based on published studies on excretion rates of virus in faeces. Our simulations show that a large variation in the viral concentration per gram of faeces between infected individuals will result in a large variability in the concentrations found in wastewater, especially when the number of shedders is low. As an example, our results show that in a hypothetical catchment area with 10 000 inhabitants and 30 persons shedding the virus daily, the expected variation on subsequent measurements of virus is large, such that 95% of the values fall within a range of 1.6 log units. This range decreases to less than 1 log unit with 300 shedders, but note that this would imply a COVID-19 prevalence larger than 3%, given that not all infected people shed the virus in their faeces. This result suggests that it will be difficult to reliably identify an increase in incidence based on wastewater surveillance data, especially if it is based on a comparison of single wastewater samples in a low-incidence situation. However, the simulations also show that with a high number of shedders and when using the mean result of a number of consecutive tests, the performance of wastewater sampling improves considerably. As the variation depends on the absolute number of shedders rather than the percentage of shedders in a catchment area, with equal incidence, the reliability of signals will be larger in large population areas than in small ones. However, sudden four- or tenfold increases in incidence may be less likely in large population areas.

Based on the need to define signals for early warning in local outbreaks, when setting up wastewater analysis as a new surveillance tool for SARS-CoV-2, the performance of potential signals was explored. Several hypothetical scenarios were defined. It was assumed that a two-, four- or tenfold increase in the number of shedders indicates a sudden increase, which is typical for an (early) outbreak or superspreading event, and should be identified by a signal. Additionally, the signal should be identified in a relatively short timeframe, to allow quick action by public health authorities. We imagined a situation where three wastewater samples are taken per week. The signal was therefore based on *k* = 3 and *k* = 6 samples, where the current 1- or 2-week period was compared with the preceding period. To identify a fourfold increase in the number of shedders, the results show that the signal-detection performs best with a signal based on *D* > 0.3 log increase in the mean number of gene copies. For a tenfold increase this is *D* > 0.6. As expected, test performance is best if the number of shedders (*N*) and the numbers of samples compared (*k*) are large.

Our simulation modelling approach is in many ways similar to that applied by others [1, 12, 28, 34]. However, by considering the log increase in concentrations and in the number of shedders over time, we did not need to describe the absolute number of gene copies or the difference in water flow between specific wastewater treatment plants, which may be sensitive to unique characteristics of the sampling method and the wastewater treatment plant. At the same time, our approach gives the possibility to identify potential outbreaks by detecting instantaneous increases in the number of shedders. We specifically applied the model to study the performance of potential signals for the detection of early outbreaks at a local scale. This is a highly relevant application of wastewater surveillance of SARS-CoV-2, as COVID-19 is expected to stay endemic, and the detection of re-emergence of the virus may not be the main purpose of the surveillance.

Our results suggest that it will be challenging to apply wastewater surveillance to detect early-stage outbreaks in a low-incidence situation at a local scale. These results may seem to contrast the many promising findings in relation to wastewater surveillance during the COVID-19 pandemic [2, 5, 6, 11, 14, 18, 35]. However, most of these authors refer to a situation where the incidence is high and/or populations contributing to the collected wastewater are large. At the other hand, studies at institutional scale typically involve smaller populations than those referred to in our study [17, 21, 22]. In these cases wastewater surveillance has shown to be an effective tool to detect the re-emergence of the virus, after it had been eliminated. Our specific purpose, however, was to identify increases in prevalence in a low-prevalence situation and not the detection of re-emergence, in populations where this could be relevant, i.e. those that fall between the small populations of concern at institutional scale and large populations considered in many other studies. The challenge of the analysis of wastewater data from smaller communities in low-incidence situations has been addressed by [36], who indicate that, in such situations, the high day-to-day variance is a key challenge for the interpretation of wastewater surveillance data. Others [37] found that, with 10 individuals shedding SARS-CoV-2 in a catchment of 100 000 individuals, there was a high likelihood of detecting viral RNA in wastewater.

For illustration, we show the performance of a few potential signals in some very specific scenarios, where the increase of the number of shedders occurs instantly. These scenarios are not realistic, as increases would often be gradual, and not exactly between two periods in which measurements are taken. The scenarios can be considered examples for which signals are most easily identified, and therefore the performance estimates are probably too optimistic. Still, superspreading events with sudden strong increases in prevalence may occur as well [38]. For these events, which may have been driving the COVID-19 pandemic [39], wastewater surveillance is expected to give clear signals.

The performance measures ‘sensitivity’ and ‘specificity’ refer to the expected rate of true positives and true negatives and provide the probability of a correct test result given the occurrence (or not) of an outbreak, defined as a two-, four- or tenfold increase in the number of shedders. For a decision maker who is mostly concerned about taking unnecessary action, it will be more important to know the positive predictive value, i.e. the probability of the occurrence of an outbreak, given that you get a signal. As explained in Appendix A, this probability depends on the rate in which outbreaks occur. If it is low, as in an endemic situation with low prevalence, the probability that a signal correctly identifies an outbreak is expected to be low, even if sensitivity and specificity are high.

Several simplifying assumptions have been made in our modelling approach. As other authors [1, 12, 28], we assume that the daily wastewater sample results do reflect the daily shedding of the virus in a homogeneously mixed wastewater system and that daily samples of the faecal mass (*fl_i_*) and the viral concentration in the faeces (*C_faeces,i_*) are independent. Additionally, we assume a proportionality of incidence and number of shedders and do not include the decline in viral concentration in the faeces that is observed over time [28, 31, 40, 41]. The assumption that changes in concentration over time reflect the changes in incidence may not be correct, especially in the situation when the incidence is decreasing and the shedding of virus continues. However, as the signals are meant to identify increases rather than decreases, the importance of this potential shortcoming is expected to be limited [42].

The analysis of the impact of the standard deviations shows that the individual variation in the viral amounts being shed largely impacts the performance of signals, especially when the number of shedders is low. Although the available data [12, 31] suggest that this variation is large, it is not well characterised. To our knowledge, it is not specifically known, neither for symptomatic *vs.* asymptomatic, nor for vaccinated *vs.* non-vaccinated people. It is also unknown whether there are differences between SARS-CoV-2 variants. Collection of that type of data would be very useful to predict the performance of wastewater surveillance. PCR measurement errors are included as a small error term, *s*_PCR_ = 0.15, but additional background noise that is often found [36] and other possible sources of pre-PCR error due to laboratory processing have not been included. The alternative analysis with *s*_PCR_ = 0.3 illustrates how an increased measurement error also impacts the performance of signals. As most of our assumptions ignore several sources of variation in sampling and analysis of the RNA data, variability in sampling data is expected to be larger than in the model predictions, and therefore the model probably overestimates the performance of signals.

## Conclusions

We used a simulation modelling approach to explore the performance of wastewater analysis-based surveillance for the detection of local SARS-CoV-2 outbreaks. Although many studies have shown that wastewater surveillance is highly promising and useful both for following trends in COVID-19 infection pressure and early detection of re-emergence, the method does not seem particularly suitable for detection of local outbreaks in low-prevalence situations. Our study showed that the substantial inherent variance in viral gene copy concentrations shed by individuals infected with SARS-CoV-2 complicates this potential usage of the surveillance tool. More specifically, our model results suggest that, for example, a situation with around 100 shedders of the SARS-CoV-2 virus, at least a fourfold increase of their number and two series of at least six consecutive samples would be needed to reliably obtain a signal (i.e. with more than 95% sensitivity and specificity). This requires intensive sampling, especially if a rapid identification of a local outbreak is required. Moreover, given the simplifying assumptions made in the analysis, such as the exclusion of several sources of variation from sampling and analysis of the RNA data, the obtained performance characteristics can be considered optimistic. As the performance of the surveillance decreases with population size and the probability of a correct signal decreases with prevalence, we do not expect to be able to perform an early identification of an outbreak at a local scale, based on wastewater surveillance.

With our analysis, we have shown that modelling can be a useful tool to increase our insight in the expected results from wastewater surveillance for SARS-CoV-2. With the large amount of data becoming available, the hypotheses generated by the modelling can be studied in detail, which may allow us to verify the underlying assumptions and increase understanding and interpretation of the results obtained.

## Figures and Tables

**Fig. A1.**
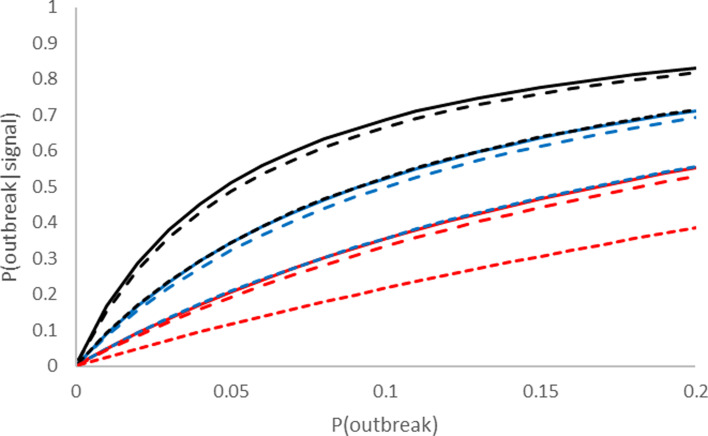
The probability that a signal is correct as a function of the outbreak occurrence rate, for different specificity (*Sp*) and sensitivity (*Se*). Black: *Sp* = 0.95, blue: *Sp* = 0.9, red: *Sp* = 0.8; straight line: *Se* = 0.99, long dash: *Se* = 0.9, short dash: *Se* = 0.5.

## Data Availability

The model used is provided as Supplementary material.

## Supplementary material

For supplementary material accompanying this paper visit http://doi.org/10.1017/S0950268823000146. click here to view supplementary material

## Appendix A The probability of a correct signal

Given that the sensitivity *Se* = *P*(signal|outbreak) and specificity *Sp* = *P*(no signal|no outbreak), with occurrence rate (outbreak probability) *P*(outbreak) = *p*, it is easy to see that the probability of a correct signal

This equation is evaluated in Figure A1, for some typical values of *Se* and *Sp*. It shows that the specificity is particularly important for the signal performance and that, when the outbreak occurrence rate is low (<<0.05), the majority of signals will be false, even with acceptable values of *Se* and *Sp*.
