# Supplementary material for: Early detection of local SARS-CoV-2 outbreaks by wastewater surveillance: a feasibility study
Source: Epidemiol Infect. 2023 Feb 1;151:e28. doi: 10.1017/S0950268823000146 (PMC9990400; doi:10.1017/S0950268823000146)
Supplement: Supplementary file 1 [file S0950268823000146sup001.docx]

Epidemiology and Infection

Early detection of local SARS-CoV-2 outbreaks by wastewater surveillance: a feasibility study

Maarten Nauta, Oliver McManus, Kristina Træholt Franck, Ellinor Marving, Lasse Dam Rasmussen, Stine Raith Richter, Steen Ethelberg

Supplementary Material'

R code for the simulation model on the performance of signals to detect outbreaks.

 setwd("C:\\path")

# concentration in feces (log10 RNA copies per g of feces)

C_fec_m <- 6

C_fec_sd <- 1

# log10 g feces shed per day

fl_m <- 2.11

fl_sd <- 0.25

#variation due to measurement uncertainty: 0.5 for measurement only; 1.0 including biological variability

pcr_unc_sd <- 0.15

#number of iterations

# with k <- 50000 it takes 2 h to run

k<-50000

# distribution of log concentrations in feces, variation per person and per day.

Cfecsample <- function (n)

rnorm(n,C_fec_m,C_fec_sd)

# distribution of log amount of feces shed, variation per person and per day.

flsample <- function (n)

rnorm(n,fl_m,fl_sd)

# risktarget indicates the % of results in my data (a list of numbers) > x,

# similar to the RiskTarget function in @Risk

risktarget <- function(mydata,x)

length(which(mydata>x))/length(mydata)

# log number of RNA copies shed with n shedders, k iterations

shedwith <-function(k,n)

{

l_ts <- vector(mode = "numeric", length = k)

for (i in 1:k)

{

C_nlog <- Cfecsample(n)

C_n <- 10^C_nlog

F_nlog <- flsample(n)

F_n <- 10^F_nlog

sheddedlog<-C_nlog+F_nlog

shedded <- 10^sheddedlog

totalshedded <-sum(shedded)

log_totalshedded <-log10(totalshedded)+rnorm(1,0,pcr_unc_sd)

l_ts[[i]]<-log_totalshedded

}

l_ts

}

# runtime control

sttime <- Sys.time()

# function to simulate k random regression analyses and difference of the mean for first n1

# and then n2 shedders, for a total of freq samplings

# In the Nauta et al. freq = 2*k, n1 is the initial number of shedders and n2 =n1*2, n1*4 or n1*10.

regranddif <- function(freq,n1,n2)

{

day3<-c(1:freq)

# number of significant regressions is put in z; z2 is for the differences of means

z <- c(1:k)

z2 <- z

for (ii in 1:k)

{

copies1 <- c(shedwith(freq/2,n1))

copies2 <- c(shedwith(freq/2,n2))

copies <- c(copies1,copies2)

# differences of means

z2[[ii]] <- mean(copies2)-mean(copies1)

# regression

regr <- lm(copies~day3)

if (summary(regr)$coefficients[2,1]>0) z[[ii]]<- summary(regr)$coefficients[2,4]else z[[ii]] <- 1

}

# outp gives a list with the input values for freq, n1 and n2 (k, N and the new N)

# and the rel. frequency in which values in z2 and z > x

outp <- c(paste(as.character(freq)," ",as.character(n1)," ",as.character(n2)),

risktarget(z2,0),risktarget(z2,0.3),risktarget(z2,0.6),risktarget(z2,0.9),risktarget(z2,1.2),1-risktarget(z,0.05),1-risktarget(z,0.1),1-risktarget(z,0.2))

new <- Sys.time()-sttime

print(paste(c(paste(as.character(freq)," ",as.character(n1)," ",as.character(n2)))," ",round(new,digits=3)))

outp

}

# here all simulations are run

allrun <- c(

regranddif(6,10,10),regranddif(6,100,100), regranddif(6,1000,1000),

regranddif(12,10,10), regranddif(12,100,100), regranddif(12,1000,1000),

regranddif(6,10,20),regranddif(6,100,200), regranddif(6,1000,2000),

regranddif(12,10,20), regranddif(12,100,200), regranddif(12,1000,2000),

regranddif(6,10,40),regranddif(6,100,400), regranddif(6,1000,4000),

regranddif(12,10,40), regranddif(12,100,400), regranddif(12,1000,4000),

regranddif(6,10,100),regranddif(6,100,1000), regranddif(6,1000,10000),

regranddif(12,10,100), regranddif(12,100,1000), regranddif(12,1000,10000)

)

ll <-length(allrun)/9

result3 <- matrix(allrun,nrow=9,ncol=ll)

#add the input values to the output file

pars<-1:ll

pars <- c(C_fec_m,C_fec_sd,fl_m,fl_sd,pcr_unc_sd,k)

for (ii in 7:ll) pars[[ii]]<-""

res4<-rbind(pars,result3)

res4

# res4 contains the values for 1-specificity and sensitivity that are written to a csv file that can be read

# e.g. in Excel to make plots.

# the first row gives the input values

# the second row gives the values for 2*k, N and increased N

# Then we get, in each column, 1-specificity (if N = increased N) or sensitivity (if increased N > N)

# for D>0; D>0.3; D> 0.6; D>0.9; D>1.2; p<0.05; p<0.1; p<0.2

write.csv(res4," scen.csv")
